# Attributing authorship via the perplexity of authorial language models

**DOI:** 10.1371/journal.pone.0327081

**Published:** 2025-07-03

**Authors:** Weihang Huang, Akira Murakami, Jack Grieve

**Affiliations:** Department of Linguistics and Communication, University of Birmingham, Birmingham, West Midlands, United Kingdom; Ariel University, ISRAEL

## Abstract

Authorship attribution is the task of identifying the most likely author of a questioned document from a set of candidate authors, where each candidate is represented by a writing sample. A wide range of quantitative methods for inferring authorship have been developed in stylometry, but the rise of Large Language Models (LLMs) offers new opportunities in this field. In this paper, we introduce a technique for authorship attribution based on fine-tuned LLMs. Our approach involves first further pretraining LLMs for each candidate author based on their known writings and then assigning the questioned document to the author whose *Authorial Language Model* (ALM) finds the questioned document most predictable, measured as the perplexity of the questioned document. We find that our approach meets or exceeds the current state-of-the-art on several standard benchmarking datasets. In addition, we show how our approach can be used to measure the predictability of each word in a questioned document for a given candidate ALM, allowing the linguistic patterns that drive our attributions to be inspected directly. Finally, we analyze what types of words generally drive successful attributions, finding that content words classes are characterized by a higher density of authorship information than function word classes, challenging a long-standing assumption of stylometry.

## Introduction

Authorship attribution is the task of identifying the author of a questioned document from a set of candidate authors, where each candidate is represented by a sample of their writing. For centuries, scholars in many fields have developed techniques to resolve cases of disputed authorship by comparing the style of a questioned document to writing samples from potential authors [1, 2]. Methods for stylometric analysis, the quantitative analysis of style, are commonly used as a general approach to resolve cases of disputed authorship [1–3]. Common approaches to stylometry involve comparing texts based on the relative frequencies of frequent words, especially grammatical function words, as well as frequent word and character n-grams, using a range of techniques for multivariate data analysis, including PCA [4, 5] and distance-based methods [6, 7].

Despite their success in certain contexts, stylometric methods for authorship attribution have clear limitations. In particular, the overall performance of these methods decreases substantially when the number of potential authors increases [3, 8], when the length of the query document decreases [9, 10], and when the amount of training data from the candidate authors decreases [8, 10]. These types of challenge greatly limit the application of stylometric methods for authorship attribution and authorship analysis more generally in the real world.

To help address these issues, recent studies have begun to draw on large language models (LLMs) for authorship analysis. Modern LLMs would appear to have considerable potential for authorship analysis because they are token-based. In traditional approaches to stylometry, linguistic features are generally defined at the type level [1, 2]. As noted above, common features in stylometry include the relative frequencies of frequent word types, like *the*, *of*, and *and*, which are measured by dividing the total number of occurrences of that word type in a text or corpus by the total number of words in that text or corpus to control for variation in text length. For each word type, a single measurement is therefore made based on all tokens of that type. Alternatively, modern LLMs are token-based [11, 12], where each individual word token is a distinct feature, allowing far more information to be extracted from any text. Compared to type-based features, token-based LLMs have proven achieve excellent performance on a wide range of text classification tasks [13]. Given that authorship attribution is a text classification task, where question texts are classified by author, it is reasonable to expect that LLM-based approaches could greatly improve performance, especially as the size of the questioned document and possible author writing samples decreases or as the number of candidate authors increases.

Examples of LLM-based methods for authorship analysis include universal authorial embeddings using Siamese BERT [14] and Character BERT [15], and using BERT for classification [16, 17]. Predictability metrics for LLMs, including perplexity and cross-entropy, have also been examined in a small number of studies. Fourkioti [18] found that the perplexity of a single LLM pre-trained on PoS-tags can be effective for authorship attribution, while Barlas and Stamatatos [19] extended this approach by training a multi-head classifier using the cross-entropy of a single pre-trained LLM. They considered several causal LLMs and masked LLM, finding that BERT achieved the best performance.

Although LLM-based approaches would appear to offer great promise for authorship attribution, their performance on standard authorship analysis tasks thus far has been disappointing. Most notably, Tyo [17] included Barlas and Stamatatos’s [19] BERT-based approach, which they referred to as pALM (per Author Language Model), in their authorship attribution benchmarking study. However, they found that pALM had the worst performance of all methods considered, which included an n-gram based classifier (Ngram) [17], a prediction-by-partial-matching compression model (PPM) [20, 21], and a pre-trained BERT model with a dense layer for classification (BERT) [16].

Alternatively, LLM-based approaches have been used with greater success in LLM detection. LLM detection involves identifying whether a questioned text was written by a human or an LLM and has gained importance in recent years due to increasing concerns about the misuse of LLMs [22–24]. Causal language model perplexity has been found to be an effective indicator of authorship [25–27], with LLM-authored texts associated with relatively low perplexity scores compared to human-authored texts. In other words, LLM-authored texts are expected to generally be more predictable for LLMs. These approaches include fully automated detection and computer-assisted detection, such as GLTR [25] and GPTZero [26].

Notably, all LLM-based approaches for authorship attribution and LLM detection have used a *single* LLM to represent candidate authors. In the case of LLM-detection, this makes sense, as the goal is to distinguish humans and LLMs in general [25, 26]. If a questioned document is highly predictable for an LLM, it is assumed to have been written by an LLM. In the case of authorship attribution, however, there are always multiple specific candidate authors, each of whom is presumed to be characterized by a unique style. For example, in standard benchmarking datasets, as we introduce below, there are often upwards of 50 different candidates to distinguish between. In such cases, measuring the predictability of a questioned document for a single LLM cannot be used to attribute the questioned document to a single candidate. In previous LLM-based research on authorship attribution, this challenge has been addressed by adding modules representing each candidate authors to the LLM and by then training the LLM for authorship prediction. However, the poor performance of pALM [17, 19] demonstrates the inherent limitations of such approaches: it appears that authorial variation is far too complex to be captured by a single LLM.

In this paper, we therefore introduce an alternative approach for using LLMs for authorship attribution. Unlike previous approaches that work with a single LLM, we fine-tune an individual LLM for *each* candidate author by further pre-training one base model on writing samples from each candidate author. We refer to each of these fine-tuned models as *Authorial Language Models* or *ALMs*. We then attribute a questioned document to the candidate author whose model is associated with the lowest perplexity. In other words, we attribute the questioned document to the candidate whose model finds that sequence of word tokens most predictable.

The remainder of this paper is organized as follows. After introducing ALMs in more detail, we present the results of bench-marking tests on four standard datasets for authorship attribution: Blogs50, CCAT50, Guardian and IMDB62 [17]. We find that ALMs achieve state-of-the-art overall performance. In addition, we demonstrate how ALMs can be used to generate token-level analyses of question documents for any candidate author, including crucially the candidate to whom our approach attributes a questioned document, allowing for the specific tokens in a text that are the most distinctive to be identified, examined, and compared. Finally, we investigate how the performance of ALMs varies when perplexity is calculated based only on tokens from specific word classes to better understand what types of words drive the success of our method. We find that content words, especially nouns, contain more authorial information than function words, challenging a long-held assumption of stylometry, where it has been argued that function words are more informative features for authorship analysis than content words [1, 4]. Ultimately, we conclude that LLMs have great potential to improve the performance and interpretability in authorship attribution and authorship analysis more generally.

## Authorial language models

**Fig 1:**
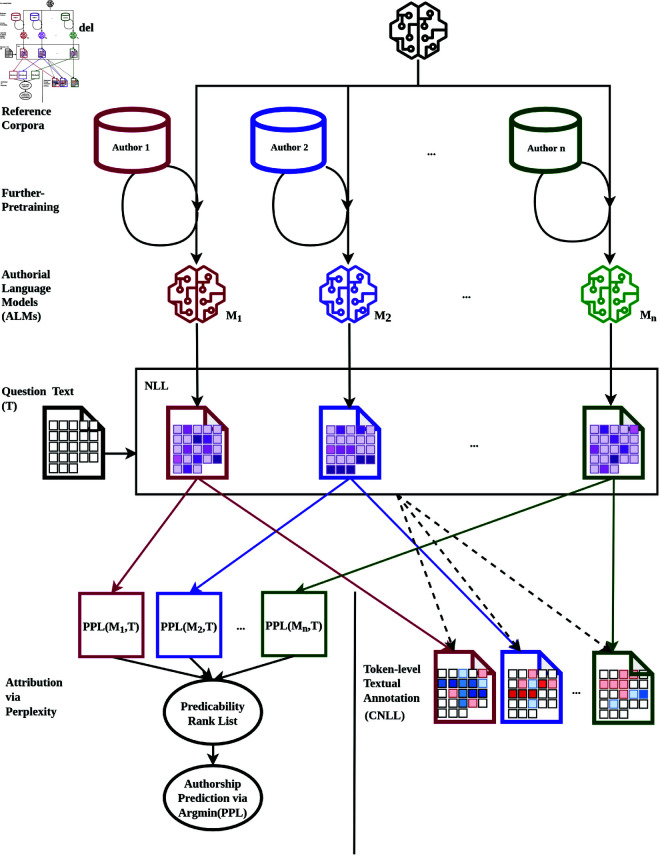
Workflow of authorial language models (ALMs).

As described above, our approach to authorship attribution follows three basic stages. First, an ALM is further pretrained for each candidate author based on a corpus of their known writings. Second, the perplexity of the questioned document is measured for each ALM and the questioned document is assigned to the candidate author whose ALM is associated with the lowest perplexity, which is the ALM that finds the questioned document the most predictable. Finally, for each ALM, word-by-word token-level predictability scores are extracted for the questioned document, which are then compared across candidate authors to identify the specific word tokens in the questioned document that are most indicative of each candidate author, especially the candidate to whom the questioned document was attributed. This allows for the attribution to be better understood, scrutinized, and explained. The ALMs workflow is shown in Fig 1, and we describe each of these steps in detail in this section.

### Further pretraining

In the first stage, we fine-tune an LLM for each candidate author by further pretraining the same base model on each candidate’s writing sample to produce a set of parallel fine-tuned LLMs that each represent the writing style of one candidate author, which we refer to as ALMs. Further pretraining is a way to update the existing parameters of a pretrained LLM based on the word sequences found in the new training texts by minimizing predictability metrics (perplexity or cross entropy) on that data, following the same basic process when pretraining a LLM from scratch [28]. In contrast to pretraining from scratch, however, where there are no existing parameters in the model, further pretraining involves fine-tuning the parameters of an existing model that were established through previous rounds of pretraining. In this way, further pretraining preserves the underlying fluency of the base model, while finely adjusting the model’s parameters so that it is better able to predict the word sequence patterns that characterize the additional pretraining data. This also crucially requires far less training data and training time than pretraining from scratch [28]. Although this approach is more efficient, it demands that we work with a base model that achieves high levels of performance out of the box. The development of transformers [29], and more specifically the decoder-only GPT model [11], provides us with a suitable base model, which had not previously been available [17].

### Attribution via Perplexity

In the second stage, we attribute the questioned document to one candidate author by measuring how predictable the sequence of tokens in the questioned document is *for each ALM* by calculating the perplexity of the questioned document for each ALM. We then assign the questioned document to the author whose ALM finds the questioned document most predictable.

The basic task of language modeling is to predict the likelihood of a word token occurring in a given context based on words that have previously been observed in context. For a causal language model, in particular, the task is to predict a word in a sequence of words based on the previous words in that sequence. Predictability metrics like perplexity are used to evaluate the ability of LLMs to predict tokens in texts and are therefore fundamental to LLM training and performance benchmarking [11]. In principle, any loss function or benchmarking method can provide information on predictability, but one of the most common predictability measurements in language modeling, especially for causal LLMs like GPT-2, is *perplexity*.

Perplexity is defined as the exponentiated negative log-likelihood of the LLM over the input text. Given a causal LLM *m*, and a text parsed as a sequence of tokens $X=\left\{x_{1},x_{2},x_{3},\ldots ,x_{t}\right\}$, the perplexity of this text over this causal LLM can be calculated as

$$\text{ppl}\left(m,X\right)=\text{exp}\left\{-\frac{1}{t}\sum_{i}^{t}\log\left({\text{p}}_{m}\left(x_{i}|x_{<i}\right)\right)\right\}$$ (1)

where ${\text{p}}_{m}\left(x_{i}|x_{<i}\right)$ is the predicted probability of the token given the preceding tokens in the sequence under the language model *m*.

A relatively low perplexity score indicates that the LLM predicts the sequence of tokens in the text with a relatively high degree of accuracy, whereas a relatively high perplexity score indicates that the LLM predicts that sequence of tokens with a relatively low degree of accuracy. To attribute the questioned document, the perplexity of the questioned document is calculated for each ALM and then the questioned document is assigned to the candidate author whose ALM is associated with the lowest perplexity score. In addition, we can rank the similarity of all candidate authors to the questioned document based on these perplexity scores.

More formally, given a set of ALMs $M=\left\{m_{1},m_{2},\ldots ,m_{n}\right\}$ that are further pretrained on texts from a set of candidate authors C={1,2,…,n}, and a questioned document *Q* from the author *i*, we calculate the perplexity (ppl) of *Q* for each ALM in *M*, which yields a set of perplexity values $\left\{\text{ppl}\left(m_{1},Q\right),\text{ppl}\left(m_{2},Q\right),\ldots ,\text{ppl}\left(m_{n},Q\right)\right\}$. Because a relatively small ppl(m,Q) indicates that *Q* is relatively predictable for *m*, $\text{ppl}\left(m_{i},Q\right)$ is expected to be the lowest among $\left\{\text{ppl}\left(m_{1},Q\right),\text{ppl}\left(m_{2},Q\right),\ldots ,\text{ppl}\left(m_{n},Q\right)\right\}$, when the author *i* is the author of the questioned document. We therefore predict that the author of the questioned document *Q* is $\text{argmin}\left(\text{ppl}\left(m_{j},Q\right)\right)$ where j∈C.

### Token-level textual annotation

In the third and final stage, we measure the distinctiveness of each word token in the questioned document for each candidate author to understand which word tokens drive the attribution returned by our system.

As Eq 1 shows, the perplexity of a text given a causal language model is calculated based on the predictability of each word token in that text for that model. For each word token in a text, the language model returns a token-level measure of predictability based on the preceding word tokens in that text, expressed as a negative log likelihood value:

$$NLL_{m,i}=-\text{log}\left({\text{p}}_{m}\left(x_{i}|x_{<i}\right)\right)$$ (2)

The sequence of token-level negative log-likelihood values for each word token in the text can therefore be analyzed to assess how predictable each word token is in the context in which it occurs for a given model. Note that NLL, as calculated in Eq 2, is subsumed by what is commonly referred to as *surprisal*, for example, in psycholinguistics [30, 31].

Specifically, given a question text *X* consisting of tokens $\left\{x_{1},x_{2},x_{3},\ldots ,x_{t}\right\}$, we record the negative log-likelihood for each token in the questioned text for each ALM in the set $M=\left\{m_{1},m_{2},\ldots ,m_{n}\right\}$, resulting in a t*n negative log-likelihood matrix:

$$\text{NLL}(M,Q)=\left(\begin{array}{c} NLL_{m_{1},1},NLL_{m_{2},1},\ldots ,NLL_{m_{n},1}\\ NLL_{m_{1},2},NLL_{m_{2},2},\ldots ,NLL_{m_{n},2}\\ \ldots ,\ldots ,\ldots ,\ldots \\ NLL_{m_{1},t},NLL_{m_{2},t},\ldots ,NLL_{m_{n},t} \end{array}\right)$$ (3)

where each row represents a token in the question text, each column represents an ALM, and each cell contains the negative log-likelihood of one token in the questioned text for that ALM.

Given a case of authorship attribution, our approach therefore not only returns the most similar author, but it also yields the predictability of each word token in the questioned document for each candidate author. However, because word tokens will generally have very similar probabilities of occurrence for all ALMs, especially because they are all derived from the same base model, the token-level predictability measurements for a single author are of limited explanatory value in this context. We therefore compare the predictability of each token across candidate authors (each row of the negative log-likelihood matrix), so as to obtain a more meaningful token-level measurement. In other words, we assess the relative predictability of each word token in the questioned document for one candidate author compared to all other candidate authors.

In the simplest case, we can compare the predictability of the *i*-th token in the text for any pair of candidate authors {a,b} by computing the difference between their negative log-likelihood values, which we refer to as pairwise *comparative negative log-likelihood* (CNLL):

$$\text{CNLL}(a,b,i)=NLL_{m_{a},i}-NLL_{m_{b},i}$$ (4)

Pairwise CNLL is effective for demonstrating the relative predictability difference between two candidate authors, because it is the difference of their negative log-likelihood at each input token for inference. When the number of candidate authors is more than two, CNLL can then be extended to *n* candidate authors by comparing the predictability of the *i*-th token in the text for one candidate author *a* to the average predictability for all other *n*-1 candidate authors in *C*:

$$\text{CNLL}(a,C,i)=NLL_{m_{a},i}-\frac{1}{n-1}\sum_{b\in \left(C\backslash \left\{a\right\}\right)}NLL_{m_{b},i}$$ (5)

These token-level CNLL values can be interpreted like text-level perplexity measurements, where CNLL(a,C,i)<0 indicates that the token *i* is relatively similar to the observed language use of candidate author *a*, compared to the other authors in *C*, because the likelihood of the word token for the ALM for author *a* is relatively high compared to the ALMs for other candidate authors. Similarly, CNLL(a,C,i)>0 indicates that the token *i* is relatively dissimilar to the observed language use of candidate author *a* compared to the other authors in *C*.

To perform a comparative token-level analysis of a questioned document for a given candidate author *m*, we can apply this method on each row of NLL(M,Q) to obtain CNLL(M,Q). The CNLL values for all tokens in a text can then be used to annotate the questioned document or to generate text-level visualizations to identify those word tokens or sequences of word tokens that provide especially strong positive or negative evidence for that author compared to the other authors under consideration. Crucially, this means that we can identify which tokens are especially distinctive for the candidate selected by our approach as the most likely author of the questioned document (or of the top *n* candidates as ranked by our approach), which are precisely those tokens that drive our attribution.

The additional information provided by this form of token-level analysis is especially important for the application of our approach in real-world cases of authorship attribution, for example, in a forensic context, where explanation and scrutiny of results is crucial for a successful attribution. This form of token-level analysis allows the results of the attribution to be more fully understood, for example, by an expert forensic linguist, both to defend and explain or to challenge and improve the attribution. An annotated questioned document could also serve as the basis for a more detailed manual analysis of stylistic variation by an expert, as well as for further computational analysis.

## Evaluation

In this section, we describe how we evaluated the accuracy of our approach to authorship attribution based on four standard benchmarking datasets [17], allowing for our method to be compared to a range of state-of-the-art methods for authorship attribution.

### Data

To evaluate ALMs, we used the Blogs50 dataset [32], CCAT50 dataset [33], Guardian dataset [17], and IMDB62 dataset [17]. We retrieved all four datasets via the scripts from https://github.com/JacobTyo/Valla. Table 1 lists the basic information for all four datasets.

**Table 1:** Dataset details.

Dataset	A	T	TK	T/A	MTL	Register
CCAT50	50	5k	2.5M	100	506	News
Blogs50	50	66k	8.1M	1.3k	122	Blogs
Guardian	13	444	467k	34	1052	News
IMDB62	62	62k	21.6M	1k	349	Film comments

**Note:** A: author count; T: text count in total; TK: token count in total; T/A: text count per author; MTL: mean text length (i.e. token count per text); Register: brief description of register of texts in the dataset.

We chose these four datasets because they are readily accessible and because they allow us to benchmark our methods directly against the methods evaluated in Tyo *et al*. (2022) [17]. In addition, CCAT50, Blogs50, Guardian, and IMDB62 each contain sufficient numbers of authors, texts, and tokens to adequately train and test language models for our method. Each dataset was also masked for URL, reference ID, character name, and other clear markers of topic that could directly conflate the task of authorship identification with topic identification [34]. The four datasets also represent different registers and present somewhat different challenges for authorship attribution.

The Blogs50 dataset consists of text from personal blogs. Notably, this dataset features relatively short text (112 tokens per text on average) that would be challenging for standard methods. In addition, the number of candidate authors is relatively large (50 authors in total), presenting additional challenges for standard authorship attribution methods. At the same time, the Blogs50 dataset contains large amounts of data for each author, approximately 1,300 texts, resulting in a total corpus size of 8.1 millions tokens, facilitating the training of ALMs.

In contrast to Blogs50, both the CCAT50 and Guardian datasets focus on news articles, and both datasets contain a smaller number of texts per author, allowing us to evaluate the accuracy of our approach on a relatively small training sample size. However, CCAT50 and Guardian are different from each other in several ways. CCAT50 is characterized by relatively short texts with an average token count of 506 words, and a relatively large number of candidate authors, equivalent to the Blogs50 dataset. On the other hand, the texts in Guardian are relatively long, with an average token of 1,052 words, which should make these texts easier to attribute, although each author is only represented by 34 texts on average, greatly limiting the amount of training data. In addition, the Guardian dataset has only 13 authors which is the smallest candidate author count among the four datasets, further reducing the relative level of difficulty of this dataset for authorship attribution.

Finally, IMDB62 consists of online comments on films. This dataset is especially challenging as it contains the largest number of candidate authors among the four datasets, with 62 candidate authors. IMDB62 also includes relatively short text with a mean text length of 349 words. In addition, IMDB62 is especially well controlled in terms of topical variation across authors compared to the other three datasets not only because every text is on the same general topic (i.e. a film review) but because in general it is extremely rare that two texts from the same author are about the same film, whereas texts from different authors can be about the same film. These challenges, however, are mitigated by the fact that this dataset is characterized by a relative large number of texts per author, and the largest corpus size in token counts (21.6 million tokens in total).

### Training

As introduced above, the first stage of our approach involves further pre-training a base model to create individual causal language models representing each of the candidate authors. The choice of the base model and fine-tuning parameters affects the performance and efficiency of our approach. Ideally, the base model should be pretrained on a large and general dataset and carefully evaluated. In addition, it is ideal if the base model is as small as possible to minimize computation costs. Given these factors, we selected GPT-2 base from OpenAI as our base model for further-pretraining. This is because GPT-2 base is a light-weighted causal language model that has been repeatedly shown to achieve high levels of performance on general datasets [11]. We fine-tuned all authorial language models with 100 epochs on one Nvidia A100 GPU. All scripts and datasets are accessible in an online repository (https://github.com/Weihang-Huang/ALMs), with key configuration values for further pre-training presented in Table 2.

**Table 2:** Further pre-training configuration variables.

Variable name	Value
Epochs	100
Learning rate	0.00002
Gradient accumulation steps	64
Weight decay	0.01

A possible concern with using GPT-2, or any LLM trained on large amounts of internet data, is that our evaluation corpora could be in the data used to train the base model. Data contamination is an issue for modern LLMs that are trained on corpora of massive size, where the test set of a downstream task is incorporated in the training corpus. In our case, however, this is not a problem. For GPT-2, in particular, we know that the data used for training was WebText, which does not appear to contain any of our training data, as described in the relevant technical reports [11]. Specifically, WebText only contains Reddit posts and related links that received at least three up-votes before 2017. Although there might be a very slight chance that a user on Reddit would copy individual texts into their comments, this would only be an extremely small portion of the corpora we used. More generally, however, our approach involves training a set of ALMs given a *single* base model. Consequently, each ALM is based on the same underlying base model, and is then fine-tuned on just the data from the one candidate. Therefore, even if the underlying base model were trained on the full evaluation corpora, this would be consistent for all ALMs: what distinguishes between the ALMs is that they were each further pre-trained on only the data from that one author. Whether the base model had also been trained on this data is irrelevant, as this influence would be consistent across ALMs given our process of parallel further pretraining.

### Accuracy metrics and procedure

Previous research in authorship attribution has used various metrics to measure performance, including f-score, precision, recall, area under the curve (AUC), and accuracy [17, 19]. Using various metrics is effective, but also makes comparison between different methods difficult. Consequently, we follow Tyo *et al*.’s (2022) approach and focus on **macro-average accuracy** as a performance evaluation metric, facilitating the comparison of the performance of our approach relative to existing methods [17]. In addition, we compute three other metrics.

First, we report **top-N macro-average accuracy**, which is an accuracy metric that treats the prediction as correct when the correct author is among the top N candidate authors. This allows us to evaluate the informativeness of our approach even when we fail to pinpoint the correct author, which becomes especially important as the number of candidate authors increases. Specifically, we investigate N∈{1,2,3,4,5}. Second, we report **true author rank** (the average rank of the correct author) as a supplementary metric. Third, we report **one versus rest accuracy for each author**, which is an author-level accuracy metric. For each candidate, we measure the proportion of correct attributions. This metric allows us to assess the performance of our approach for each of the authors individually, which is useful for assessing whether questioned documents written by certain authors are substantially more difficult to attribute.

Finally, to test the robustness of our method, we compute each of these accuracy metrics using 5 random 8:2 train–test splits on all four evaluation datasets, reporting the mean accuracy metrics as well as standard error, where the mean accuracy metrics show the performance of our method in general, and the standard error shows the estimated deviation from this average performance.

## Results

### Text-level attribution

In this section, we present the results of our evaluation of the overall accuracy of our approach for automated authorship attribution. Table 3 compares the macro-average accuracy and the standard error of the macro-average accuracy of our method compared to other state-of-the-art methods based on the Blogs50, CCAT50, Guardian and IMDB62 datasets [17].

**Table 3:** Comparison of macro-accuracy between our method (ALMs) and recent SOTA methods [17].

Method	Variable	Blogs50	CCAT50	Guardian	IMDB62	Mean
ALMs	Acc.	0.883.6	74.9	94.5	0.899.5	0.888.1
	SE	0.1	1.2	0.5	0.1	0.5
Ngram	Acc.	72.3	0.876.7	0.8100	98.8	87.0
	SE	0.4	1.5	0.0	0.0	0.5
BERT	Acc.	75	65.7	84.2	98.8	80.9
	SE	0.7	1.3	0.7	0.1	0.7
PPM	Acc.	72.2	69.4	86.3	95.9	81.0
	SE	0.6	1.4	0.7	1.0	0.9
pALM	Acc.	69.3	63.4	66.6	90.4	72.4
	SE	0.3	1.5	0.6	0.9	0.8
Random Baseline	Acc.	2	2	7.7	1.6	3.3

Overall, we find that our method outperforms all other methods under consideration, achieving a mean macro-accuracy over all four datasets of 88.1%. In addition, our method outperforms all other methods on the Blogs50 and IMDB62 datasets, achieving a macro-average accuracy of 83.6% and 99.5%, respectively. In addition, on the CCAT50 and Guardian datasets, our method outperforms all but one method, achieving a macro-average accuracy of 74.9% and 94.5%, nearly matching the Ngram method, which is the best approach on these datasets and achieves a macro-average accuracy of 76.7% and 100%. Furthermore, the standard error of the mean macro-accuracy of ALMs on all evaluation datasets only shows a negligible deviation, indicating that the performance of ALMs is robust and stable for different train-test split.

These results clearly show that ALMs can be a very accurate method for authorship attribution: in general, it meets or surpasses the accuracy of all the other state-of-the-art methods. This is especially true when working with short texts like those in Blogs50 and IMDB62 where the average questioned text lengths are limited to 122 words and 349 words, respectively. In databases with longer questioned texts such as CCAT50 and Guardian, the comparative advantage of our method over other approaches is less pronounced, but our method still achieves very high levels of performance. It is also important to note that we obtain our best overall results on the IMDB62 dataset, which is characterized not only by short texts but also by presumably greater topical balance across authors, as discussed above.

Given that our approach ranks the predictability of the questioned document for all candidate authors, we also calculated the Top-N macro-average accuracy to evaluate how well our approach performs, even when it fails to precisely attribute the questioned document. This is an especially important metric when working with a large number of candidate authors, as is the case for three of our four datasets (i.e., Blogs50, CCAT50, and IMDB62), because it can still be very informative to narrow down the number of candidates in real-world cases with many authors. Specifically, we considered N∈{1,2,3,4,5} and report these macro-average accuracy scores in Table 4, where the first column contains the same results as reported in Table 3.

**Table 4:** Comparison of Top-N macro-accuracy between our method (ALMs) and recent SOTA methods [17].

Method	Dataset	Top-1	Top-2	Top-3	Top-4	Top-5
ALMs	Blogs50	83.6	88.4	90.6	92.2	93.0
	CCAT50	74.9	85.6	90.9	92.9	94.0
	Guardian	94.5	96.8	97.7	98.5	98.5
	IMDB62	99.5	99.8	99.9	99.9	99.9
Ngram	Blogs50	72.3	78.2	83.2	85.6	86.7
	CCAT50	76.7	88.3	92.7	93.3	95.5
	Guardian	100.0	100.0	100.0	100.0	100.0
	IMDB62	98.8	99.9	100.0	100.0	100.0
BERT	Blogs50	75.0	78.2	80.3	82.1	83.7
	CCAT50	65.7	69.1	72.1	76.2	79.9
	Guardian	84.2	85.2	86.3	87.2	88.8
	IMDB62	98.8	99.1	99.4	99.9	99.9
PPM	Blogs50	72.2	74.4	76.9	78.2	81.2
	CCAT50	69.4	74.2	75.7	78.3	79.2
	Guardian	86.3	87.1	87.3	87.4	87.5
	IMDB62	95.9	96.4	97.2	97.7	97.9
pALM	Blogs50	69.3	71.5	74.2	76.9	78.0
	CCAT50	63.4	67.2	71.0	73.3	74.1
	Guardian	66.6	68.2	68.8	69.2	69.3
	IMDB62	90.4	91.6	91.9	92.3	92.5

The results show that our method achieves a macro-accuracy of 90% in all datasets at Top-3 and 93% at Top-5. In other words, even when working with 50 or more candidate authors, we can generally be confident that the correct author will almost always be among the five most similar candidates returned by our system for the datasets we have analyzed. Compared to other methods, at Top-5, only ALMs achieves a macro-accuracy over 90% on all four datasets, especially on Blogs50 and IMDB62.

In addition, we measured the rank of the true author in all evaluations, including the mean, standard deviation, and the top 25%, 50%, 75%, and 99% percentile of the true author rank, as reported in Table 5. We find that our method achieves the correct prediction at Top-1 for the easiest 50% of the questioned documents in all four datasets, and for Blogs50, Guardian and IMDB62, this percentage is even higher (75%). Moreover, at Top-2, our method achieves the correct prediction for 75% of the questioned documents. Finally, for the most difficult 1% of the questioned documents, our method still ranks the correct author in the first 60% percentile of cases, even for the most challenging dataset (CCAT50).

**Table 5:** Comparison of true author ranks between our method (ALMs) and recent SOTA methods.

Method	Dataset	Mean	STD	Q25	Q50	Q75	Q99
ALMs	Blogs50	2.1	4.1	1	1	1	24
	CCAT50	2.3	4.8	1	1	2	29
	Guardian	1.2	0.9	1	1	1	6
	IMDB62	1.0	0.2	1	1	1	1
Ngram	Blogs50	2.5	4.7	1	1	2	20
	CCAT50	1.9	3.9	1	1	1	8
	Guardian	1.0	0.0	1	1	1	1
	IMDB62	1.0	0.2	1	1	1	1
BERT	Blogs50	3.6	4.9	1	1	1	32
	CCAT50	4.2	5.1	1	1	4	35
	Guardian	1.6	1.2	1	1	1	9
	IMDB62	1.1	0.3	1	1	1	1
PPM	Blogs50	4.3	4.8	1	1	3	25
	CCAT50	4.7	4.9	1	1	3	24
	Guardian	1.4	1.1	1	1	1	9
	IMDB62	1.2	1.1	1	1	1	7
pALM	Blogs50	4.6	4.8	1	1	3	37
	CCAT50	5.2	5.6	1	1	5	41
	Guardian	5.7	5.8	1	1	8	25
	IMDB62	1.6	1.2	1	1	1	14

**Note:** STD: standard deviation; Q25: top 25% percentile; Q50: top 50% percentile; Q75: top 75% percentile; Q99: top 99% percentile.

Finally, to evaluate the robustness of our method among individual authors, we calculated single author accuracy scores for the Blogs50 (see Table 6), CCAT50 (see Table 7), Guardian (see Table 8) and IMDB62 (see Table 9) datasets. For 38 of the 50 authors in Blogs50, 31 of the 50 authors in CCAT50, 12 of the 13 authors in Guardian, and the 62 authors in IMDB62 we obtained an accuracy of more than 80%. However, we note that there are a few authors whose texts prove especially difficult to attribute, such as Author 46 in Blogs50 and Author 30 in CCAT50.

**Table 6:** Performance of our method for each of the 50 authors in Blogs50.

Author name	Text #	Token #	Mean text length	Accuracy (%)	SE
0	2623	306575	116.9	90.9	0.2
1	1405	341521	243.1	94.9	0.1
2	1311	298754	227.9	86.9	0.2
3	1301	207581	159.6	83.7	0.1
4	1215	254051	209.1	96.1	0.1
5	1207	194987	161.5	92.4	0.1
6	1125	144410	128.4	86.5	0.2
7	1100	119820	108.9	95.6	0.2
8	1083	257212	237.5	83.4	0.1
9	1078	182824	169.6	89.6	0.1
10	1046	456278	436.2	88.2	0.1
11	1019	166745	163.6	43.5	0.1
12	1009	164719	163.2	73.4	0.1
13	947	132354	139.8	88.2	0.1
14	910	191351	210.3	91.7	0.2
15	894	211436	236.5	95.1	0.2
16	835	237192	284.1	80.9	0.1
17	830	162396	195.7	84.1	0.1
18	811	288834	356.1	95.1	0.1
19	808	63683	78.8	99.5	0.1
20	807	160624	199	91.6	0.1
21	795	132298	166.4	84.9	0.2
22	782	290040	370.9	90.8	0.1
23	755	331710	439.4	83.1	0.1
24	753	91592	121.6	83.5	0.2
25	743	213729	287.7	76.3	0.2
26	740	102774	138.9	67.6	0.1
27	740	148279	200.4	98.9	0.1
28	726	111685	153.8	83.4	0.1
29	726	96883	133.4	75.1	0.2
30	718	87430	121.8	88.3	0.1
31	716	499373	697.4	97.8	0.2
32	707	128940	182.4	76.3	0.2
33	706	181419	257	88.6	0.2
34	705	118039	167.4	80.7	0.1
35	689	79639	115.6	83.7	0.1
36	661	87402	132.2	64.2	0.1
37	636	203631	320.2	98.7	0.1
38	631	359944	570.4	93	0.1
39	621	248852	400.7	67.1	0.2
40	609	50015	82.1	82.2	0.2
41	609	128077	210.3	88.2	0.1
42	605	81499	134.7	75.5	0.2
43	605	90913	150.3	84.8	0.1
44	605	456833	755.1	96	0.1
45	600	78737	131.2	50	0.1
46	593	86792	146.4	39.2	0.1
47	592	132766	224.3	60.8	0.1
48	576	202813	352.1	94.4	0.1
49	565	118027	208.9	95	0.1

**Table 7:** Performance of our method for each of the 50 authors in CCAT50.

Author name	Text #	Token #	Mean text length	Accuracy (%)	SE
0	90	55807	620.1	50.0	0.8
1	90	63795	708.8	68.0	1.6
2	90	65540	728.2	66.0	1.4
3	90	62633	695.9	88.0	1.0
4	90	63459	705.1	96.0	1.5
5	90	62785	697.6	88.0	1.6
6	90	58634	651.5	90.0	1.2
7	90	51316	570.2	82.0	0.5
8	90	59730	663.7	30.0	1.5
9	90	57452	638.4	88.0	0.8
10	90	57416	638	92.0	1.4
11	90	54029	600.3	40.0	0.8
12	90	61247	680.5	60.0	1.2
13	90	58148	646.1	34.0	0.6
14	90	53918	599.1	80.0	1.8
15	90	53871	598.6	82.0	1.6
16	90	65597	728.9	60.0	1.5
17	90	65092	723.2	30.0	1.3
18	90	53021	589.1	84.0	1.3
19	90	70496	783.3	80.0	1.6
20	90	57850	642.8	78.0	1.0
21	90	64731	719.2	94.0	1.3
22	90	68547	761.6	80.0	1.5
23	90	61034	678.2	88.0	1.0
24	90	69984	777.6	100.0	1.0
25	90	56861	631.8	92.0	0.7
26	90	55312	614.6	90.0	1.6
27	90	66168	735.2	68.0	1.0
28	90	52956	588.4	86.0	1.2
29	90	57617	640.2	86.0	2.0
30	90	65783	730.9	14.0	0.9
31	90	43818	486.9	82.0	1.7
32	90	53929	599.2	90.0	1.5
33	90	59234	658.2	60.0	1.2
34	90	66891	743.2	28.0	1.7
35	90	63705	707.8	100.0	0.6
36	90	59393	659.9	80.0	1.4
37	90	64213	713.5	78.0	1.6
38	90	53000	588.9	62.0	1.6
39	90	58239	647.1	48.0	0.7
40	90	54635	607.1	86.0	0.4
41	90	68160	757.3	82.0	1.2
42	90	56512	627.9	98.0	1.5
43	90	63766	708.5	92.0	1.2
44	90	62480	694.2	42.0	0.9
45	90	56889	632.1	92.0	1.3
46	90	57731	641.5	72.0	1.1
47	90	55562	617.4	100.0	1.0
48	90	67006	744.5	88.0	1.2
49	90	41518	461.3	100.0	1.4

**Table 8:** Performance of our method for each of the 13 authors in Guardian.

Author name	Text #	Token #	Mean text length	Accuracy (%)	SE
0	11	16454	1495.8	100.0	0.3
1	10	14404	1440.4	100.0	0.5
2	10	12243	1224.3	100.0	0.8
3	11	16216	1474.2	100.0	0.4
4	10	15732	1573.2	100.0	0.4
5	9	9802	1089.1	100.0	0.5
6	12	17493	1457.8	100.0	0.5
7	11	14698	1336.2	90.9	0.5
8	9	11712	1301.3	88.9	0.5
9	9	10940	1215.6	88.9	0.4
10	10	7413	741.3	60.0	0.3
11	9	13096	1455.1	100.0	0.5
12	10	14224	1422.4	100.0	0.5

**Table 9:** Performance of our method for each of the 62 authors in IMDB62.

Author name	Text #	Token #	Mean text length	Accuracy (%)	SE
0	200	49065	245.3	100.0	0.0
1	200	20849	104.2	99.0	0.1
2	196	64005	326.6	99.5	0.0
3	200	74902	374.5	99.5	0.1
4	200	154296	771.5	100.0	0.0
5	200	82235	411.2	98.5	0.1
6	200	31904	159.5	98.0	0.0
7	200	80360	401.8	97.5	0.1
8	200	38501	192.5	99.0	0.1
9	200	137208	686	99.0	0.1
10	200	30252	151.3	99.5	0.1
11	200	59123	295.6	100.0	0.1
12	200	158347	791.7	99.0	0.0
13	200	35416	177.1	96.5	0.0
14	200	39564	197.8	99.5	0.0
15	200	31293	156.5	100.0	0.1
16	200	154300	771.5	99.5	0.1
17	200	39220	196.1	99.0	0.1
18	200	46488	232.4	99.5	0.0
19	200	66781	333.9	100.0	0.0
20	200	54908	274.5	100.0	0.1
21	179	38996	217.9	100.0	0.1
22	200	44585	222.9	99.0	0.1
23	200	107014	535.1	97.5	0.1
24	200	85523	427.6	100.0	0.1
25	200	67338	336.7	100.0	0.1
26	200	157838	789.2	99.5	0.1
27	200	74844	374.2	99.5	0.0
28	198	104431	527.4	99.5	0.1
29	200	82713	413.6	100.0	0.0
30	200	86571	432.9	100.0	0.0
31	200	67508	337.5	100.0	0.1
32	200	141492	707.5	100.0	0.1
33	200	39919	199.6	98.0	0.0
34	200	98346	491.7	100.0	0.1
35	200	101012	505.1	100.0	0.0
36	200	44413	222.1	97.0	0.1
37	200	102560	512.8	100.0	0.1
38	200	63959	319.8	100.0	0.1
39	200	86262	431.3	99.0	0.1
40	200	60466	302.3	100.0	0.1
41	200	47063	235.3	100.0	0.1
42	200	62954	314.8	100.0	0.1
43	198	67258	339.7	100.0	0.1
44	200	105740	528.7	99.5	0.0
45	200	155525	777.6	100.0	0.1
46	200	68725	343.6	100.0	0.1
47	200	61358	306.8	100.0	0.1
48	200	78057	390.3	100.0	0.1
49	200	106664	533.3	100.0	0.1
50	200	173509	867.5	100.0	0.1
51	200	70725	353.6	100.0	0.1
52	200	63312	316.6	100.0	0.1
53	200	58586	292.9	99.5	0.1
54	199	68634	344.9	99.0	0.1
55	200	46371	231.9	99.5	0.0
56	200	90194	451	100.0	0.1
57	200	85598	428	100.0	0.1
58	200	134946	674.7	100.0	0.1
59	200	51904	259.5	99.5	0.1
60	200	44550	222.8	100.0	0.0
61	200	59644	298.2	100	0.1

### Token-level textual annotation

In addition to producing a list of candidates ranked by the predictability of the questioned document, our approach also annotates the questioned document at the token level for any candidate, showing how predictable each token is for that author. This information complements the attribution. Most importantly, when applied to the top candidate (or to each of the top *n*-candidates), it directly reveals which word tokens drove the perplexity-based attribution returned by our system.

**Fig 2:**
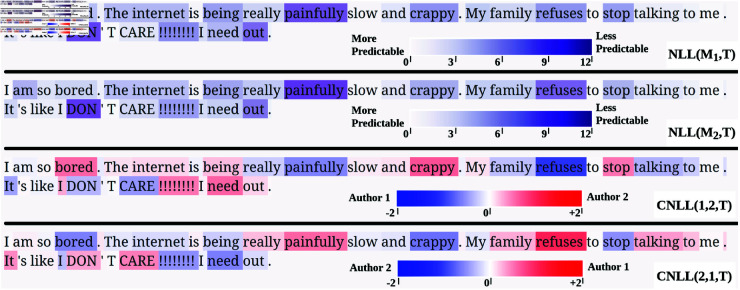
Token-level text annotation: Text 26-18 in the Blog50 dataset (top-2 authors).

To illustrate this process, we begin with the simplest case, which involves a pair of candidate authors. In Fig 2, we present token-level NLL predictability scores, calculated independently for each author, for the top two candidates for text 26-18 in the Blogs50 dataset. Our approach correctly attributes this questioned document to the first candidate. The first annotated text presents the NLL scores for the first author, while the second annotated text presents the NLL scores for the second author, where color saturation represents the unpredictability of that token given that author’s model.

As can clearly be seen in Fig 2, the NLL annotations for the two authors look very similar. This is because in general the same words are approximately equally predictable for both ALMs, reflecting patterns instantiated in the shared base model, which represents something like the English language in general [35]. Rather, what we want to know is how predictable each token is for a given author compared to the other candidates under consideration. We therefore convert these NLL scores into CNLL scores by comparing the token-level NLL scores for both authors (see Eq 4), which are presented in the third and fourth annotated texts in Fig 2, where blue represents CNLL<0, indicating evidence in favor of the first candidate author and against the second candidate author, red represents CNLL>0, indicating evidence against the first candidate author and for the second candidate author, and color saturation represents the strength of this evidence in either direction. Note that because we are only considering two authors, the fourth annotated text in Fig 2 is simply the inverse of the third annotated text.

Examining the third annotated text in Fig 2, we can now clearly see which individual word tokens are more strongly associated with the first candidate author as opposed to the second. For example, we can see that the use of the word *refuses* is especially indicative of the first candidate. Other words that are indicative of the first candidate include the tokens *painfully*, *talking*, and *CARE*. Alternatively, words whose usage is more consistent with the second candidate include *crappy* and the second *I*, although ultimately this negative evidence is outweighed by the positive evidence for the first candidate.

**Fig 3:**
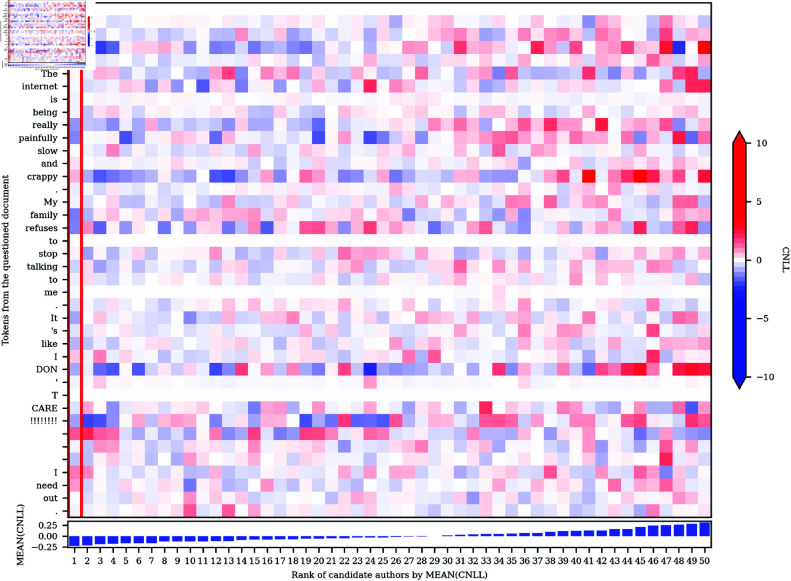
CNLL heatmap: Text 26–18 in the Blogs50 dataset.

**Fig 4:**
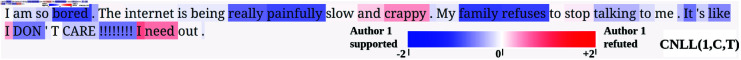
Token-level text annotation: Text 26-18 in the Blog50 dataset (top author).

We can also extend the CNLL analysis to any number of candidates (see Eq 5). Most importantly, we can compare the token-level NLL values for our selected candidate to the mean token-level NLL values for all other candidates, identifying the specific word tokens that drive any attribution returned by our approach. For example, in Fig 3, we plot the CNLL matrix for all tokens in text 26-18 in the Blogs50 dataset for all 50 candidates, where each row represents one token in the questioned document, and where each column represents one candidate author, ordered based on the attribution ranking returned by our system. Accordingly, the author selected by our system (in this case, correctly) is found in the first column, which is highlighted by the red rectangle. Once again, blue represents CNLL<0, indicating evidence in favor of that author, and red represents CNLL>0, indicating evidence against that author, but in this case compared to all the other candidates, as opposed to just one. For the sake of clarity and comparison with Fig 2, we also present the CNLL scores for only the selected author for each token in the questioned document in a textual format in Fig 4.

Examining the heatmap in Fig 3, we can now see why our system attributes the questioned document to the selected author rather than to any of the other 49 candidates: the token-level CNLL values for that candidate are, on the whole, lower than they are for the other candidate authors. Visually, there is more blue and especially, in this case at least, less red in the first author’s column. In other words, on average, the word tokens found in the questioned document are more predictable by the correct author’s model, with very few words being especially unpredictable for that author’s model compared to the other 49 authors under consideration. This is reflected by the relatively large difference in the mean CNLL value for the selected candidate and all other candidates, as presented in the graph at the bottom of Fig 3, which naturally are highly correlated with perplexity.

A more detailed inspection of Fig 3 allows us to identify the specific tokens that provide the strongest evidence for a given candidate, as well as counter evidence, but now compared to the other 49 candidates as opposed to just one. By looking down any column of this heatmap we can see the evidence for or against any candidates compared to the other candidates. Most importantly, the column for the selected author shows precisely how much each token contributed to their selection over the other 49 candidates. In this case, the tokens that are especially indicative of the selected author include *bored*, *really*, *painfully*, *family*, *refuses*, *It*, and *DON*, whereas the tokens that provide counter-evidence include *crappy*, the second and third tokens of *I*, and *need*. Looking across rows of the heatmap, we can see that some word tokens are more distinctive than others, i.e. rows with many strongly shaded red

and blue cells. For example, the presence of *crappy* is clearly, perhaps unsurprisingly, a very marked word for many authors, being much more predictable for some authors than others, moderately unexpected for our selected author, although not enough to shift our attribution.

If we compare Fig 4 to the third annotated text in Fig 2, we can see that the distinctiveness of tokens can change as we change the number of candidate authors. Most notably, *bored*, which was assigned a strong positive CNLL score in the 2-author comparison, indicating evidence against the selected author, was assigned a strong negative CNLL score in the 50-author comparison, indicating evidence for the selected author. Looking at the second column of Fig 3, we can see that this token still (necessarily) provides even stronger evidence for the second-ranked author, but this usage of *bored* is, nevertheless, still more indicative of the selected author than most of the other 48 candidates.

Fig 3 also illustrates how different tokens of the same word type can provide evidence for and against different candidate authors. For example, the first token of *I*, in the first position of the text, is approximately equally predictable for all authors, whereas the predictability of the second and especially the third token of *I* are much more variable across authors, as well as being relatively inconsistent with the writing style of the selected author. Crucially, this result contrasts clearly with traditional stylometric analyses, where linguistic variation is generally measured based on the token counts of word types, so that all tokens of the same type are combined to form a single measurement, precluding analysis that varies at the token level.

More generally, as illustrated by the first token of *I*, as well as the first token of *to*, *me*, and *T*, it is clear that some tokens are not distinctive for any author under consideration, as shown by a predominantly white row, which indicates that these tokens are approximately equally likely for all candidates. These tokens therefore do not contribute one way or another to our attribution. In general, these are words that are highly predictable in the given context. For example, the use of infinitive *to* following the verb *refuses* is clearly a very common pattern in the English language. Even the token *T*, which is part of the capitalized negation of the verb *do* (i.e., *DON*), is very predictable for all authors even though *DON* is not, as presumably some authors are more likely to use capitalization in this way for emphasis. However, following DON and an apostrophe, these models all reasonably find capital *T* in the next position to be highly predictable for all authors, as would be expected.

In addition to word tokens, our approach also allows for authorial information to be extracted from punctuation marks and other non-lexical patterns, which are often ignored in traditional stylometric analyses. For example, the four periods in this text are of variable predictability across our candidate authors, although these tokens are relatively indicative of our selected author. Similarly, the heatmap also shows how authorial information can be extracted from patterns in whitespace, in this case the three consecutive breaks that follow *CARE*. As is the case for word tokens, there is clear variation across these three breaks: while the first line-break token is unexpected for our selected author, the final line-break token is judged as being indicative of their style, presumably reflecting a tendency of this author to stack up line breaks, although, looking across that row of the heatmap, we can see this pattern is much more strongly associated with other candidates.

Overall, we can therefore clearly see how informative the type of token-level analysis produced by ALMs can be for understanding and explaining the patterns driving attributions. It also illustrates how our approach is similar to the type of qualitative *stylistic* authorship analysis often conducted in a forensic context, where linguists examine a questioned document one word at a time [36, 37]. Forensic analysts work at the token level not only because this is how we read a text, but because it allows more evidence to be drawn from the texts under analysis, which can often be very limited in number and length. This is also the great advantage of ALMs, as the analysis of token-level annotations illustrates.

## The stylistic significance of content words

In addition to the applied value of ALMs for resolving cases of disputed authorship, many of the token-level analyses we have considered during our evaluations have revealed a surprising pattern: content words (e.g., *refuses*, *painfully*, *talking*) appear to carry more authorial information than function words. When we look at the word tokens that are associated with the lowest CNLL scores for a selected candidate, and which therefore drive the attributions returned by ALMs, we find that they tend to come from content word classes like nouns, adjectives, and verbs, as opposed to function word classes like prepositions, conjunctions, and determiners.

This observation is so surprising because it would appear to be at odds with one of the most basic assumptions in the field of authorship analysis: as we have noted, the use of function words has long been considered to be a better marker of authorship than the use of content words. The supremacy function words was most notably a major theme in Mosteller and Wallace’s 1963 study of the *Federalist Papers*, one of the foundational texts of stylometry. The basic problem with content words, according to Mosteller and Wallace, is that their use depends on the topic as opposed to the style of a text (2007: 265): “We need variables that depend on authors and nothing else. Some function words come close to this ideal, but most other words do not. So many words and other variables depend on topics that their exploration for differences between authors would be a needless waste of funds.” This basic claim has since been re-stated many times [1–5, 38–43]. To take a recent example, Nini (2024: 2) writes that Mosteller and Wallace’s “insight survived the test of time and to this day the analysis of word frequencies and especially of the frequencies of function words is still among the most successful ways of carrying out authorship analysis.”

Although it is indisputable that the use of content words depends at least in part on the topic of a text, given our informal observation that content word tokens often appear to be highly indicative of authorship, we decided to follow up our main benchmarking study with a post-hoc analysis designed to evaluate the general contribution of word tokens from different word classes to the overall accuracy of ALMs.

We first tagged each word token in the texts used for evaluation with their word class, using part-of-speech (PoS) tags from the universal tag set [44]. This process is complicated by the fact that the tokenizer used for GPT-2, the BPE Tokenizer, does not align with how most PoS taggers would tokenize a text, leading to misalignment. To address this issue, we performed PoS tagging on the corpus following BPE tokenization. Although this slightly lowers PoS tagging accuracy, it ensures alignment between PoS tags and tokens. We selected the SpaCy English transformer (en_core_web_trf [45]) pipeline for PoS tagging, with its tokenizer overridden by the BPE tokenizer.

For each word class, we then recomputed our benchmarking accuracy scores for ALMs: rather than measuring the overall perplexity of a text (*X*) given a model (*m*) based on the predictability of *all* word tokens in that text 1, we only considered word tokens from one word class (*F*) at a time.

$$\text{fppl}\left(m,X,F\right)=\text{exp}\left\{-\frac{\sum_{i}^{t}\text{log}\left({\text{p}}_{m}\left(x_{i}|x_{<i}\right)\cdot [x_{i}\in F]\right)}{\sum_{i}^{t}[x_{i}\in F]+1}\right\}$$ (6)

In Eq 6, the square brackets are Iverson brackets, which take the value 1 when the statement within is true and 0 otherwise. We refer to this value as **filtered perplexity**, as it is a measure of perplexity that only considers the NLL of word tokens that have been tagged as belonging to a specific word class.

**Fig 5:**
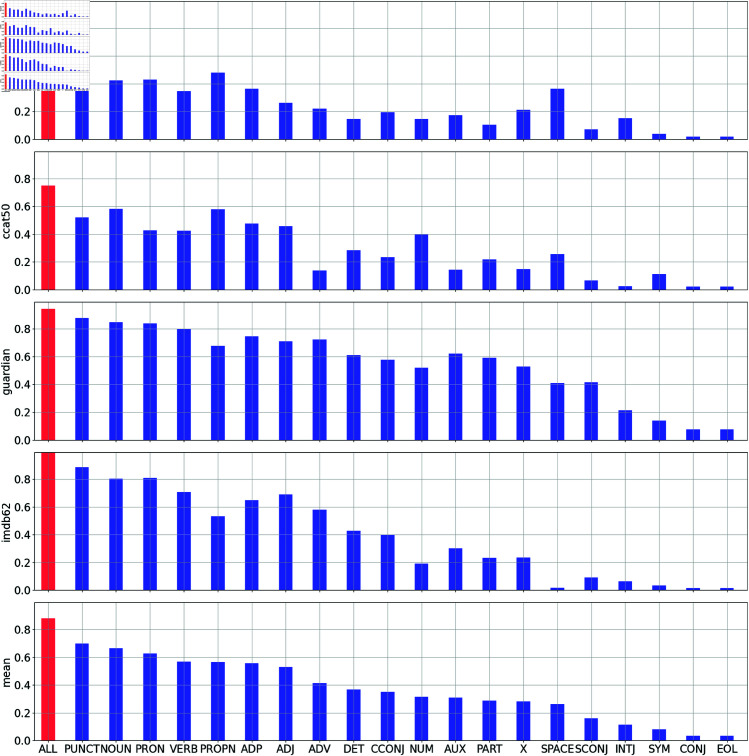
Macro-accuracy of filtered perplexity for POS tags.

Fig 5 shows the macro-accuracy scores of ALMs with filtered perplexity applied to 20 word class tags in the Blogs50, CCAT50, Guardian, and IMDB62 datasets. These scores are represented by blue bars. For comparison, we also plot the macro-accuracy scores of ALMs using unfiltered perplexity as red bars, replicating the results from our main evaluation. Although we see that the best results are obtained when when word tokens from all word classes are taken into consideration, maximizing the amount of information used in attribution, there is considerable variation in which word classes are most informative on average.

These results clearly show that our informal observations are correct: content words are more informative than function words when attributing authorship using ALMs. Overall, Nouns (NOUN), Verbs (VERB), Proper Nouns (PROPN), and Adjectives (ADJ) are in the top 7 of all 20 classes analyzed, with Nouns in second place after Punctuation Marks (PUNCT), which are also outside the standard stylometry feature set. That is not to say that function words do not provide authorial information: when filtered on any relatively common word class, ALMs achieves accuracy scores far above random chance. Furthermore, as we have noted, we obtain the best results when all word classes are included in the analysis. Some function word classes are also relatively informative, especially Pronouns (PRON) and Prepositions (ADP), although it is notable that pronouns are often excluded from function word analyses because they are seen as being too dependent on topic [4, 38]. Nevertheless, it is clear, across all four datasets, that content word tokens are consistently more indicative of authorship than function word tokens – very much at odds with received wisdom.

**Fig 6:**
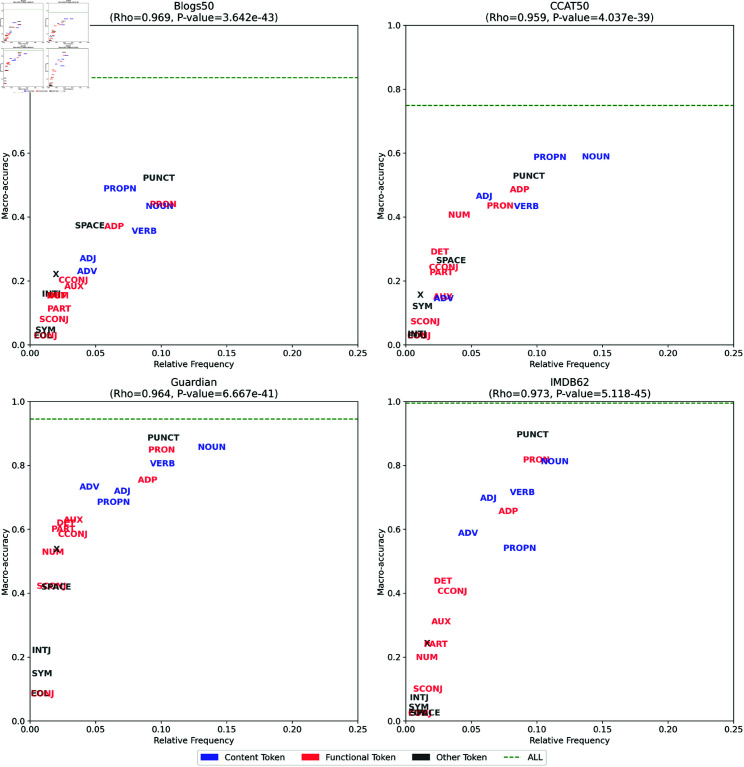
Scatteplots of frequency and macro-accuracy via ALMs using filtered perplexity for each dataset.

Before we consider why this is the case, it is important to note that these filtered accuracy scores are closely related to the frequencies of the word classes being filtered on: the more frequent a word class, the better the results we obtain when calculating perplexity based only on those tokens. To test this observation, for each dataset, we calculated Spearman’s correlation coefficient between the frequency of each word class and the macro-accuracy of ALMs when based only on word tokens from that class. We found that this correlation was very strong, exceeding 0.95 for the four datasets, as can be seen in Fig 6.

It is not the case, however, that nouns and other content word classes are relatively good markers of authorship *because* they are relatively frequent: filtered perplexity is normalize based on the number of matching tokens, as can be seen in Eq 6, where the sum of the filtered log likelihood values is divided by the number of matches. In other words, it is not the case that nouns are especially informative just because they are very frequent, with each noun token contributing a relatively small amount of information to the analysis. Rather, we find that nouns *on average* carry more authorial information.

We hypothesize that content words are more informative than function words when working at the token level because there is much greater diversity within content word classes. As a result, there is greater potential for variation in the choice between content words compared to function words by an author at any point in a text. While function word classes are composed of very small numbers of word types, content word classes are composed of very large numbers of word types. The selection of a content word *in a given context* therefore has the potential to convey far more authorial information than the selection of a function word in a given context, which can likely only be one of a small number of words. In fact, in many contexts, only one function word is really possible, as shown, for example, by rows entirely populated by white cells in our CNLL heatmaps, which are generally found only for function words.

**Fig 7:**
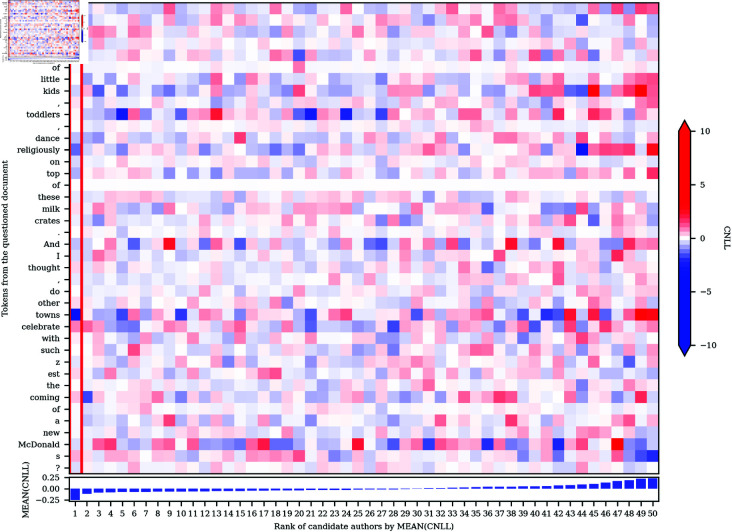
CNLL heatmap: Text 47–18 in the Blogs50 dataset.

To better appreciate how much stylistic information can be carried by a single content word, in this case a single noun, consider Fig 7, where we present the CNLL heatmap for all 50 authors for text 47–18 in the Blogs50 dataset, which was once again correctly attributed by our approach. It is notable that the difference in the mean CNLL values between the selected author and all other candidates is especially large, indicating a very definitive attribution. Accordingly, if we look down the column for the selected author, which is once again highlighted by a red rectangle, several highly indicative words are apparent, including *remember*, *religiously*, and especially *towns*, which largely drives this attribution.

The word *towns* is an unexpected token of authorship, as it would seem to be a word type, like most nouns, that is closely related to a specific topic, and therefore not likely to be indicative of authorship. Nevertheless, we find that this token of the word *towns* in this text is very indicative of authorship. From a technical standpoint, this is possible because our analysis is token-based as opposed to type-based. In other words, this result does not amount to a claim that the (frequent) use of the word type *towns* is *generally* indicative of this author, but rather that this singular use of the word token *towns* in this specific context is indicative of this author compared to the other candidates under consideration. The value of content words has been underestimated because this form of evidence cannot be captured through traditional type-based analyses: although it seems reasonable to assume that the relative frequency of content word types in a text will largely reflect the topic of that text, we find that the use of individual content word tokens can be highly indicative of authorship.

But why then is this token of *towns* so distinctive? This is not a text about *towns*, although it is a text about a new fast-food restaurant coming to a specific town. Rather, what seems to be especially interesting about the use of the word *towns* in this text is how it is being used *discursively* – not to reference that town, but to metonymically reference people from other towns in general so as to allow the author to indirectly express an opinion about the possible uniqueness of the behavior of the people who live in this particular town through comparison, for rhetorical and comedic effect. In what way exactly this author’s complex rhetorical strategy in this sentence, which is centered on their use of the word *towns*, is unique compared to the other candidates under consideration would require a detailed discursive analysis of the writing samples under analysis. But this example illustrates how a token of a single content word can contain a great deal of stylistic information, which is highly indicative of authorship.

## Discussion

In this paper, we have introduced a new LLM-based method for authorship attribution that we refer to as *Authorial Language Models* or *ALMs*. Our basic approach involves training a language model for each candidate author by fine-tuning a single base model on each author’s known writing. We then assign a questioned document to the candidate whose model finds that document most predictable based on the perplexity of that document computed for each author’s model. In addition, we have shown how our perplexity-based metric can be decomposed so that we can analyze the predictability of each word token in the question document for a selected author compared to the other candidates. In this way, the patterns of linguistic variation that underlie our attributions can be inspected directly. Our evaluations found that ALMs meet or exceed the state-of-the-art in authorship attribution on a number of standard bench-marking datasets, covering a range of registers and topics. Our approach achieves high accuracy levels especially in cases with large numbers of candidate authors and short questioned documents.

Compared to other recent studies that have introduced techniques for authorship analysis based on LLMs, the primary methodological innovation of ALMs is that we fine-tune an *individual* language model for each candidate author, and then assess how consistent the language of a questioned document is with that model via perplexity. Furthermore, compared to conventional stylometric methods, the primary methodological innovation of ALMs is that we analyze texts at the *token* level. In contrast to type-based approaches, where all instances of a given word type are combined to create a single measurement point for a text (e.g. the relative frequency of that type in that text), a token-based approach, which is made possible by using LLMs, treats each token of a word type as a distinct measurement point, thereby offering the potential for far more information to be extracted from each text than is possible using type-based approaches, moving beyond analyzing word frequency [10, 46]. Furthermore, these differences account for the success of our approach compared to standard stylometric techniques, especially when working with short questioned documents, which have always posed a major challenge for authorship analysis [10].

The power of a token-based approach to authorship attribution becomes especially clear when considering the results of our token-level annotations, which allow us to directly inspect the specific tokens whose use drives our attributions. Notably, we find that not all tokens of the same word type are equally informative: even when they occur in the same questioned document, tokens of the same word type can be more or less indicative of a given author, depending on the context in which they occur, which is precluded by type-based analysis.

We also find that content words, as opposed to function words, which have long been the focus of stylometric analysis, generally contain substantially more information about authorship. Content words have been excluded from stylometric analysis because they have been assumed to depend more on the topic of a text than on its style. This would appear to be true when working at the type-level; however, because ALMs involves analyzing texts at the token-level, content words can be used as indicators of authorship. In this way, our approach allows us to potentially extract authorial information from a far large larger proportion of word tokens in any text than traditional stylometric techniques, which we believe accounts for the accuracy of our approach, even when working with short texts. From a theoretical perspective, this is also an especially surprising result, challenging the long-standing assumptions that the usage of function words is a better indicator of authorship than the usage of content words, further highlighting the value of working at the token level.

Given the methodological and theoretical insights working with this approach has provided, there are several clear and important directions for future research. At the most basic level, it is important to test our approach on more diverse and challenging corpora, including corpora representing a wider range of registers, dialects, and languages. In general, a major potential advantage of our approach is that there is no need for feature selection or complex grammatical analysis, making our method suitable for attributing authorship in any language, given a base model for that language. In addition, although we have found ALMs perform well with short questioned documents and a large number of candidates, compared to other state-of-the-art methods, it is especially important to consider how much the performance of ALMs depends on the amount of training data available for each candidate author. In all four datasets, the amount of data per author is substantial, whereas in real-world cases of disputed authorship, the amount of training data can be quite limited.

There are also various technical modifications to our basic paradigm that can be tested. For example, although we have found that calculating perplexity based on all word tokens yields substantially better results than filtering by any individual word class, we have not yet tested more complex filters (e.g., filtering on all content words), which could yield better results. Similarly, we could also test the effect of varying our approach to word tokenization, especially as the default tokenization used by LLMs – in this case the Byte Pair Encoding (BPE) algorithm [11]—can be quite different from how we might otherwise tokenize a text, for example, splitting words we might prefer to keep as single tokens. This could be especially useful for improving the interpretability of our results.

In addition, it is important to test our general approach using other causal LLMs. In this paper, we have worked with the GPT-2 base model, which is characterized by a relatively small number of parameters compared to more recent models. Working with larger models could potentially improve our attributions, although it is important to acknowledge that using an LLM with a larger number of parameters would have a substantially larger footprint in terms of computation and memory usage. Nevertheless, even using GPT-2, ALMs were generally able to achieve state-of-the-art performance.

It would also be informative to consider how the performance of our approach changes when based on other types of LLMs. One reason we have focused on causal LLMs is the simplicity of their predictability metrics. Crucially, perplexity, as calculated in Eq 1, is specific to causal LLMs. Consequently, interpreting NLL as a predictability ’building block’ is also restricted to causal models. Although causal LLMs are very popular, not all models define context based only on preceding tokens. For instance, masked language models like BERT and RoBERTa consider both preceding and following tokens and have also been used for authorship analysis [16]. Similarly, pre-transformer neural networks define contexts and predictability in different ways and have been used for authorship analysis as well [47, 48]. Using non-causal models within our general ALMs framework would require a more general approach to measuring predictability. We believe token-level cross entropy could serve as a general alternative to NLL, since token-level cross entropy is a general measurement of the predictability of one probability distribution compared to another, regardless of the type of language model that is used and how context is defined.

In addition to these directions for future technical research, we are also especially interested in exploring the applicability of ALMs to help resolve real-world cases of disputed authorship. We believe the form of token-level CNLL annotation generated by our approach is especially useful in real-world applications, as it provides a basis for scrutinizing the attributions returned by our system, including summarizing, explaining, and disputing these attributions, for example, by an expert forensic linguist. Furthermore, an annotated questioned document can provide a basis for directing further textual analysis by an expert. In a forensic or security context, where expert analysis is often critical, analysis is greatly limited by the amount of time it takes to read through large amounts of textual data to identify features of interest. However, as we have illustrated in this paper, annotating a text with CNLL values can be very informative and could act as a map for an expert analyst, directing their focus to the most distinctive parts of the text.

Finally, we believe that the method introduced in this paper for authorship analysis can also be used for comparative corpus linguistics more generally. Given corpora that represent varieties of language of interest [35], we can fine-tune language models that represent each variety. For example, such models can represent not only authorial varieties (i.e. idiolects), but dialects (e.g. British, American, and Canadian English) and registers (e.g., fiction and nonfiction writing). Given additional texts drawn from these corpora, fine-tuned models can then be used to measure the distance between the varieties and to discover features that are distinctive of these varieties. Moving forward, we therefore believe that our general approach has widespread application for linguistic analysis, allowing for varieties of language to be described, compared, and classified in a replicable, holistic, and data-driven manner, with far-reaching implications for empirical linguistic research.
